# Bon-Bons

**Published:** 1906-05

**Authors:** 


### Bon-Bons.

"The 'Red Back' gives us the stuff we need."—R. S. MARTIN.

MEDICINE is not a rigid system of rules and formula as it was in ancient Egypt; a fixed creed to which you are to subscribe, and from which you must not vary. It is a living, growing thing, making use of every resource which the progress of science brings; testing all things and holding fast to that which is good.—***Dr.*** *John S. Billings' Address to the Graduating Class, Belvue Medical College, 1882.*

spring Lake, ju 2

Mr editur sir pleas Send Me A Samp Copey of You danial med jour with a View of subscribing i am taking 4 & o Bligfrat B Horst m. D

SUMMER SESSION, BY THE LECTURERS AND ASSISTANTS, NEW ORLEANS POLYCLINIC.—This course is intended for recent graduates and other physicians who have been unable to attend earlier. It will last six weeks, and begin May 21, 1906. Teaching in eighteen branches, including the specialties, laboratory work and cadaveric operations. Table of rates: Any single branch, six weeks, $15; four weeks, $12; any two or more branches, each six weeks, $12; four weeks, $10; all branches, six weeks, $100; four weeks, $75. For further particulars, write New Orleans Polyclinic, Liberty and Tulane Avenue, New Orleans, La.

---

HERE IS A GEM, worth preserving by all lovers of the wittiest and bravest and most patient and long-wronged race of men on earth; and, although it in no way relates to medicine, I give it to my readers, who, I am sure, will appreciate it. It appeared in the Dallas *News*, St. Patrick's Day in the morning, March 17, 1906:

RIGHT THE WRONGS OF IRELAND.

Come all who boast o' Gaelic blood—
O'Brien, Burke, Fitzgerald, Flood;
The hour is near at hand
When England and her Parliament
Must right the wrongs of Ireland.

Eight hundred years and more we've fought—
Years with untold suffering fraught;
Died many a patriot band
To force the Tyrant and his lords
To right the wrongs of Ireland.

Let Leinster, Munster and Connaught,
And Ulster, too, by deed and thought,
March ever hand in hand.
Let all unite to pray and fight
To right the wrongs of Ireland.

Then will the time be near, indeed,
When Celts o' every clan and creed
In glorious union stand.
Then shall the friend of all oppres'd
Make right the wrongs of Ireland.

—*Peel Morphy Payne, M. D., Quinlan, Texas.*

THE next regular meeting of the State Board of Medical Examiners will be held in Dallas, Texas, June 12, 13, 14.

T. T. JACKSON, Secretary,
San Antonio, Texas.

---

THE first commencement exercises of the State Dental College, Dallas, Texas, were held on the evening of May 4th at Carnegie Hall. The address of the evening was delivered by the Hon. E. B. Muse, of the city of Dallas; invocation by Rev. Thornton Whaling, D. D. The degree of D. D. S. was conferred upon George Robert Smith, Horace Overby, Chas. Curtis Baker, and Kanematsu Taniuchi, and the degree of D. M. D. was conferred upon Thos. Glyndon Bradford, D. D. S.

---

THE BULLY BRAZOS VALLEY MEDICAL ASSOCIATION will meet at Hearne May 29th and 30th. This is an unattached, unreconstructed, and unterrified district association, and holds together with great tenacity and unflagging interest. Dr. H. S. Coulter, Rockdale, is president; Dr. A. S. Epperson, Rockdale, is secretary pro tem., in place of the lamented Briggs.

---

PUEBLO, MEXICO, May 2, 1906.

*Dr. F. E. Daniel, Austin, Texas.*

DEAR DOCTOR: Please find enclosed check for $2 (*dos pesos*), to cover my account with the best State medical journal, edited by the biggest-hearted doctor I know of in two Republics.

T. H. HARRELL, M. D.

---

THE "RED BACK."—New subscribers in two days: Drs. White, Vernon, Texas; Garrett, Springtown, Texas; Risinger, Roscoe, Texas; Smith, Burgess, Texas; Gregory, Big Sandy; Strand, Henderson, Texas; Yates, Kirbyville, Texas; Alf. Irby, Weatherford, Texas; Calloway, Anson, Texas; Redford, Boyd, Texas; Lawrence, Longview, Texas; Ramsay, Joaquin, Texas; Spivey, Joaquin, Texas; Moseley, Dallas, Texas; Ward, Blum, Texas; Keyes, Rockwall, Texas; Amman, Turnersville Texas; Westbrook, Sipe Springs, Texas; Hall, Fort Worth, Texas; Dobbins, Troy, Texas; Wier, Beaumont, Texas; Payne, Roger's Prairie, Texas; Hill, Galveston, Texas; Miller, Hillsboro, Texas; Goddard, Halland, Texas; Raines, Mineral Wells, Texas; Cocke, Mineral Wells, Texas; S. O. Moore, Winnsboro, Texas; Scott, Houston, Texas; Millen, Elm View; Ervin, McKinney, Texas;

Montgomery, Ryan, I. T.; Phillips, Chase, Texas; Howard, Ashland, Texas; Richards, Ashland, Texas; Morrison, Grosvenor, Texas; Pope, Kountze, Texas; Burns, Gainesville, Texas.

---

A HANDSOME BACTERIOLOGICAL WALL CHART, in colors, for physicians' office will be sent free to any of my readers upon request, mentioning the "Red Back." It is scientifically accurate and highly artistic and ornamental.

---

SKILL.—"We congratulate you upon having made the best display of our latest advertisement copy that we have yet seen. It looks well on the front page of the 'Red Back.' Will you kindly send us two or three proofs printed on good paper so that we can keep your display as our type sample. Very truly yours,

"SHARP & DOHME, N. Y."

---

Y. M. D.'S SOLILOQUY.

I would write for Daniel's red JOURNAL,
Such papers as ne'er were seen,
If it wouldn't expose how infernal
Incompetent I am—and how green:—
My pieces I'd make scientific,
And prepare with elaborate pains,
They'd be read e'en to the Pacific,
But hang it! I haven't the brains.

---

CONSISTENCY.—We like to hear people speak out bravely with the courage of their convictions. Even though they may be wrong, it is yet better that they speak bravely, for only by such free speech can the truth come to be known. There is nothing to be expected from the coward and the liar. W. C. ABBOTT.

---

Salisbury suggests that drugs be given to be absorbed from the mucous membrane of the mouth, for which the simple alkaloid is better fitted than cruder forms. The intercepting action of the liver is thus avoided and smaller doses will accomplish the desired results. Digestion will not be injured and nausea produced. The paper closes with some remarks on untoward effects of some drugs upon the stomach, and circumstances delaying absorption. — *Am. Jour. Clin. Med.*

Fond Mother.—"Yes, doctor, the boy's got a cough on him like a bar'l. Cough for the doctor, Johnny."

---

Must Have Something Hot.—Dear Dr. Daniel: Please send my Journal to Elk City, Okla., instead of to Cheyenne, as heretofore. As this is the best town in Western Oklahoma, I must have something *hot* (red) to mix with it. Fraternally,

Elk City, Okla., May 7, 1906. J. E. Standifer.

---

THE DEAD BEAT AND THE DOCTOR.

VERY ILL.

Name, oh doctor, name your fee,
Ask—I'll pay whatever it be—
Skill like yours, I know comes high,
Only do not let me die.
Get me out of this and I
Cash will ante instantly.

CONVALESCENT.

Cut, oh doctor, cut that fee,
Cut, or not a cent from me.
I am not a millionaire,
But I'll do whatever's square,
Only make a bill that's fair,
And I'll settle presently.

WELL.

Book, oh doctor, book your fee,
Charge, I'll pay it futurely;
Whenever the crops all by are laid,
When every other bill is paid,
Or when of death again afraid,
I will pay it—probably!

---

### From The Forks of The Creek.

Gentry, Tenn., April 19, 1906.

*Dr. Daniel, Editor "Red Back," Austin, Texas.*

Dear Doctor: I am a little obscure country doctor living in the hills of Tennessee, far removed from any public thoroughfare, doing all my practice horseback, carry my own medicine, being a specialist of diseases of women and children, eye, ear, nose, and throat; genito-urinary and general surgery, and special attention being given to internal medicine, and also an expert tooth puller. In fact, I am such an adept at my business that I am classed as a combined animal, and known in the medical fraternity as a general practitioner of medicine. I never appear in society, only occasionally at some medical meeting, and the toot of a railroad engine scares me nearly to death. Not given to boasting, but I have a very lucrative practice, neither gold nor silver, but the necessities of life. My pay consists in razor-back and sand-digger swine, chickens, corn, Irish potatoes, promises, etc. In order to enhance my income I stand a bull and a boar, which enables me to pay for the "Red Back"; and right here I want to thank you deep down in my heart for the editorial on "Simmons and the American Medical Association," in the April number. While I am only 37 years old, being born since the Civil War, and know nothing of the horrors of the Civil War, if Simmons keeps stuffing the "nigger" down our throats, then why not have another "Missouri Compromise" and a re-establishing of the old "Mason and Dixon" line, and a secession of all Southern doctors in an association of their own, letting it be known far and wide that we are the "Lily Whites," and no patent medicine man, osteopath, eclectic, homoeopath and no "nigger" is admitted into the sanctum. If Simmons believes in amalgamation, either physically or professionally, let him have it, but let us be sure no "coons" sup at the communion with Southern M. D.'s.

Though born and reared in Tennessee, I have always been partial to Texas doctors, so keep after Simmons with a hot iron until the nigger is exiled. My olfactory has already perceived the stench arising from the "coon" at the annual banquet in Boston in June.

SCOTT FARMER, M. D.

---

### The American International Congress on Tuberculosis.

The officers of this Congress announce its next session for November 14, 15, and 16, 1906, at the city of New York.

The published call is as follows:

OFFICE OF THE PRESIDENT,
AUSTIN, TEXAS, February, 1906.

The Executive Officers of the American International Congress on Tuberculosis, with the approval of its Governing Council, announce that the body will hold a Congress in the city of New York, commencing the 14th day of November, 1906, to be held three days.

All the officers, members and delegates are invited to attend, and to contribute papers to be read at the Congress, and to send the title of papers to the Secretary as early as possible.

This Congress will be open to members of all the professions, legislators, statesmen, the intelligent laity and the reverend clergy.

An enrolling fee of $3 is solicited to defray the expenses of a Bulletin, which should be sent forthwith to the Treasurer.

All the governments in the western hemisphere are invited to send delegates to this Congress and to co-operate in its labors.

The public press, lay and medical, are hereby invited to give publicity to this announcement.

F. E. Daniel, President,
Austin, Texas.
Matthew M. Smith, Secretary,
Austin, Texas.
Clark Bell, Esq., LL. D., Treasurer,
139 Broadway, New York.

The foregoing announcement and call for the session of the American International Congress on Tuberculosis as signed by the Executive Officers, is hereby approved by a majority of the Governing Council and Board of Executive Officers.

MEMBERS OF THE COUNCIL.

Dr. J. Mount Bleyer, of New York City.
Dr. N. K. Foster, State Board of Health, Sacramento, Cal.
Dr. A. P. Grinnell, 500 Fifth Avenue, New YorkCity.
Dr. Denslow Lewis, Chicago, Ill.
Dr. Andrew C. Smith, President State Board of Health, Portland, Ore.

The following officials have signed their names to the foregoing call, or authorized the Secretary or Treasurer to sign for them:

HONORARY AND EX-PRESIDENTS.

E. J. Barrick, M. D.
A. N. Bell, M. D.

VICE AND EX-VICE PRESIDENTS.

Chas. Wood Fassett, M. D., of St. Joseph, Mo.
Chas. K. Cole, M. D., New York City, late of Montana.
A. M. Linn, M. D., Des Moines, Ia.

Hon. L. Bradford Prince, Santa Fe, N. M.
N. O. Nelson, Esq., St. Louis, Mo.
Dr. Sofus B. Nelson, Pullman, Wash.

EX-MEMBERS OF THE COUNCIL.

Moritz Ellinger, New York City, Chairman.
M. K. Kassabian, M. D., Philadelphia, Pa.
R. J. Nunn, M. D., Savannah, Ga.
W. F. Drewry, M. D., Petersburg, Va.
J. W. P. Smithwick, LaGrange, N. C.
T. D. Crothers, M. D., Hartford, Conn.
J. C. Shrader, M. D., Iowa City, Ia.

VICE-PRESIDENTS-AT-LARGE.

T. D. Crothers, M. D., Hartford, Conn.
I. C. Shrader, M. D., Iowa City, Ia.
Louis LeRoy, M. D., Nashville, Tenn.
Prof. J. H. Tyndale, M. D., Lincoln, Neb.

VICE-PRESIDENTS OF STATES.

Alfred E. Regensburger, M. D., San Francisco, Cal.
Sophie Hinzie Scott, M. D., Des Moines, Ia.
Mary J. Ardery, M. D., Knoxville, Tenn.
Wm. Lee Howard, M. D., Baltimore, Md.
Geo. W. Grover, Nashua, N. H.

This call is intended to be addressed to every member of the American International Congress on Tuberculosis, and to each and every delegate accredited to the Congress since its organization, in February, 1900, and to all citizens interested in the conflict for the suppression of tuberculosis. It will be sent especially to all boards of health and organizations against tuberculosis in the western hemisphere, including State, provincial, and county medical organizations, who it is hoped will send three delegates from each body and advise us of names and addresses.

The Governors of the various States whose names are now on its lists of honorary vice-presidents are requested to give this notice publicity, both in the States, Territories, and dependencies of the American Union, also the executives of all the governments in the western hemisphere, with the Governors of the States and provinces therein, are requested to give this announcement publicity, so that it may reach men of science in all those countries interested in the conflict with tuberculosis, so that they may co-operate in the great problem of preventive legislation against tuberculosis, municipal and governmental sanitariums, and all the questions involved in the struggle with the great scourge of the race.

The Bulletin of the Congress of 1904 is now ready and has been sent to subscribers. So long as the edition lasts it will be sent on application to the *Medico-Legal Journal* on receipt of $2. It is a large volume of 476 pages, with a large number of illustrations. If any subscriber has not received the Bulletin he is requested to apply for it at once.

The *Medico-Legal Journal* will send a copy of Vols. 22, 23, and 24 to any member or delegate to this Congress at half price, $1.50, if paid in advance.

Members and delegates who desire to enroll will remit the enrolling fee of $3, and will be entitled to receive the Bulletin of the Congress free, if enrolling fee is sent to the Treasurer, Clark Bell, Esq., 39 Broadway, New York. Members of the organization who do not wish to enroll can retain their place as members on the payment of the annual dues of $1.

Contributions to defray the expenses of the Congress are solicited by the executive officers.

OFFICERS.

F. E. Daniel, M. D., President, Austin, Texas.
Matthew M. Smith, M. D., Secretary, Austin, Texas.
Clark Bell, Esq., LL. D., Treasurer, 39 Broadway, New York.

VICE-PRESIDENTS.

1st—Dr. J. H. Kellogg, Battle Creek, Mich.
2d—Dr. Charles Wood Fassett, St. Joseph, Mo.
3d—Dr. John Ferguson, Toronto, Ontario.
4th—Hon. H. O. Nelson, Edwardsville, Ill.
5th—Hon. D. Pat Dyer, St. Louis, Mo.

COUNCIL, 1905-1906.

Dr. P. Oliver Hanford, Chairman, Colorado Springs.
Dr. J. Mount Bleyer, New York City.
Dr. N. K. Foster, Sacramento, Cal.
Dr. A. P. Grinnell, New York City.
Dr. Denslow Lewis, Chicago, Ill.
Dr. H. Edwin Lewis, Burlington, Vt.

Dr. W. F. Morrow, Kansas City, Mo.
Dr. Andrew C. Smith, Portland, Ore.
Dr. Edmond Souchon, New Orleans, La.

VICE-PRESIDENTS-AT-LARGE.

Daniel Clark, M. D., Toronto.
Charles G. Hicks, M. D., Dublin, Ga.
Wm. Bayard, M. D., St. John, N. B.
H. Edwin Lewis, M. D., Burlington, Vt.
A. C. Bernays, M. D., St. Louis, Mo.
J. Mount Bleyer, M. D., New York City.
Juan A. Fortich, M. D., Cártagena, Colombia, S. A.
R. F. Graham, M. D., Greely, Colorado.
A. P. Grinnell, M. D., 500 Fifth Ave., New York City.
Capt. H. D. Snyder, M. D., Governor's Island, N. Y.
Thos. Bassett Keyes, M. D., Chicago, Ill.
Luis H. Labayle, M. D., Leon, Nicaragua.
Dr. Louis LeRoy, Nashville, Tenn.
Dr. Edouard Liceaga, City of Mexico.
Dwight S. Moore, M. D., Jamestown, N. Dak.
Rev. C. S. Eby, D. D., Bracebridge, Ont.
Dr. W. F. Morrow, Kansas City, Mo.
Dr. John H. Simon, Health Commissioner, St. Louis, Mo.
Col. E. Chancellor, M. D., St. Louis, Mo.
Wm. F. Brunner, M. D., Savannah, Ga.
T. D. Crothers, M. D., Hartford, Conn.
Hon. Moritz Ellinger, New York City.
Dr. R. W. Powell, Ottawa.
Wm. H. Murray, M. D., Plainfield, N. J.
Ex-Sen. Henry D. Winton, Hackensack, N. J.
J. A. McNeven, M. D., Gibbonsville, Idaho.
A. E. Osborne, M. D., Santa Clara, Cal.
J. C. Shrader, M. D., Iowa City, Iowa.
Prof. J. H. Tyndale, M. D., Lincoln, Neb.
T. L. Barber, M. D., Charleston, W. Va.
C. S. Ward, M. D., Warren, Ohio.
Prof. S. H. Weeks, M. D., Portland, Me.
Dr. W. H. Moorehouse, London, Ont.
Dr. W. P. Cavan, Toronto.

### HONORARY VICE-PRESIDENTS.

*From the States, Territories, and Dependencies of the American Union.*

Alaska—Hon. John G. Brady, Ex-Governor, Sitka.

Arizona—Hon. N. O. Murphy, Ex-Governor of the Territory.

California—Hon. George C. Pardee, Sacramento; Dr. A. E. Regensberger, San Francisco.

Colorado—Hon. James H. Peabody, Denver.

Georgia—Hon. L. E. Bleckley, Ex-Chief Justice, Atlanta; Hon. Allen D. Candler, Atlanta.

Idaho—Hon. Frank W. Hunt, Ex-Governor, Boise City.

Iowa—Hon. Albert B. Cummins, Ex-Governor; Dr. Sophie Hinzie Scott, Des Moines; Dr. Mary D. Ardery, Knoxville; Dr. J. C. Shrader, Iowa City.

Kansas—Hon. W. E. Stanley, Ex-Governor, Topeka.

Kentucky—Hon. J. C. W. Beckham, Ex-Governor, Frankfort.

Maine—Hon. John F. Hill, Ex-Governor, Augusta.

Maryland—Dr. Wm. Lee Howard, Baltimore.

Michigan—Hon. Aaron T. Bliss, Ex-Governor, Lansing.

Minnesota—Hon. S. R. Van Sant, Governor, St. Paul.

Mississippi—Hon. A. H. Longino, Ex-Governor, Jackson.

Missouri—Hon. Alex. M. Dockery, Ex-Governor, Jefferson City.

Montana—Hon. Joseph K. Toole, Ex-Governor, Helena.

Nebraska—Hon. E. T. Savage, Ex-Governor, Lincoln; Prof. J. H. Tyndale, M. D., Lincoln.

New Hampshire—Hon. C. B. Jordan, Ex-Governor, Ludlow; Dr. George W. Grover, Nashua.

New Jersey—Hon. Franklin Murphy, Ex-Governor, Trenton.

New Mexico—Hon. Miguel A. Otero, Ex-Governor, Santa Fe; L. Bradford Prince, Santa Fe.

North Carolina—Hon. Charles B. Aycock, Ex-Governor, Raleigh.

Pennsylvania—Hon. Wm. A. Stone, Ex-Governor, Harrisburg.

Rhode Island—Hon. Lucius F. C. Garvin, Ex-Governor, Providence.

South Carolina—Hon. M. B. McSweeney, Ex-Governor, Columbia.

South Dakota—Hon. Charles N. Herried, Ex-Governor, Pierre.

Tennessee—Hon. Benton McMillin, Ex-Governor, Nashville.

Texas—Hon. Joseph D. Sayers, Ex-Governor, Austin.

Utah—Hon. Heber M. Wells, Ex-Governor.

Vermont—Hon. William W. Stickney, Ex-Governor, Montpelier.

Vrginia—Hon. A. J. Montague, Ex-Governor, Richmond.

Washington, D. C.—Hon. David J. Brewer, U. S. Supreme Court.

West Virginia—Hon. A. B. White, Ex-Governor, Charleston.

Wyoming—Hon. Deforest Richards, Ex-Goxernor, Cheyenne.

Cuba—Gen'l. Leonard Wood, M. D., Military Governor, Philippines.

HONORARY VICE-PRESIDENTS.

*Fom the Dominion of Canada.*

Hon. A. G. Blair, Ex-Minister of Railways and Canals, Ottawa.

Hon. William S. Fielding, Minister of Finance, Ottawa.

W. C. Edwards, M. P., President Canadian Association for the Prevention of Tuberculosis, Ottawa.

Dr. F. Montizambert, Director of the Public Health, Ottawa.

*From the Provinces of the Dominion of Canada.*

British Columbia—Hon. Henry G. Joly de Lotbiniere, Lieut. Governor, Victoria.

New Brunswick—Hon. J. Bunting Snowball, Lieut. Governor, St. John, N. B.

New Foundland—Hon. Sir Cavendish Boyle, Colonial Governor, K. C. M. G., St. John.

North West Territories—Hon. A. E. Forget, Lieut. Governor, Regina.

Nova Scotia—Hon. A. G. Jones, Lieut. Governor, Halifax.

Ontario—Lieut. Governor Hon. W. Mortimer Clark, Toronto.

Quebec—Hon. A. Robitaille, Provincial Secretary, Quebec; Dr. T. G. Roddick, M. P., Montreal, Quebec; Sir William Hington, M. D., Montreal, Quebec; Hon. Senator Geo. A. Drummond, Montreal, Quebec; James Loudon, President of the University of Toronto; Hon. J. R. Stratton, Provincial Secretary, Toronto.

VICE-PRESIDENTS.

*From the States of the American Union.*

Alabama—W. H. Blake, M. D., Sheffield; Dr. R. M. Cunninghom, Ensley; John C. LeGrande, M. D.

Alaska—Hon. Governor C. D. Rogers, Juneau; Dr. Park Winter Stewart, Nome City.

Arkansas—Dr. H. C. Dunavant, Little Rock.

Arizona—John C. Herndon, Esq., Attorney-at-Law, Prescott; Dr. Jones Tempe, Prescott.

California—Alfred E. Regensburger, 14 Grant Avenue, San Francisco; Hon. A. Reuf, San Francisco; Dr. P. C. Remondino, San Diego; Dr. N. K. Foster, Secretary Board of Health; Dr. Winslow Anderson, San Francisco; Dr. S. M. Pottinger; Prof. Norman Bridge, Los Angeles.

Colorado—Dr. Charles C. Rice, Pueblo.

Connecticut—Hon. James P. Bree, State Senator.

Delaware—Dr. W. S. Hancken, Farnhurst.

Florida—J. Harris Pierpont, M. D., Pensacola; D. J. DeWitt Webb; R. D. Murphy, Key West.

Georgia—Hon. T. B. Fetter; Dr. H. McHatton, Macon; Dr. Arthur A. Van Dyke, Atlanta; Charles Hicks, Dublin; C. C. Jones, M. D., President State Medical Society.

Hawaii—W. O. Smith and C. B. Cooper, M. D., of Honolulu; B. D. Bond, Esq., Kohala.

Idaho—Dr. J. B. Morris, Lewiston; Dr. William F. Smith, Mountain Home; Mr. Charles L. Joy, Boise City.

Illinois—Dr. Denslow Lewis, Dr. W. E. Quine, Chicago; Hon. N. O. Nelson, Edwardsville; Dr. Henry B. Favill, Chicago; S. C. Fairbrother, East St. Louis.

Indiana—Dr. George T. McCoy, Columbus; Flavius J. Van Vort, Esq., Indianapolis; T. Henry Davis, Richmond; W. B. Fletcher, Indianapolis.

Iowa—J. J. Gibson, M. D., Ex-State Veterinary Surgeon, Denison; Hon. J. M. Emmert, M. D., State Senator, Atlantic; Dr. Jennie McCowen, Davenport.

Kansas—W. E. McVey, M. D., Topeka.

Kentucky—Prof. Frank C. Wilson, M. D., Louisville.

Louisiana—Dr. Felix Fermento, 135 Esplanade Ave., Prof. Dr. J. B. Elliott, Morris Building, Dr. P. G. Archinard, all of New Orleans.

Maine — Prof. F. N. Whittier, M. D., Brunswick; Dr. A. G. Young, Augusta; Dr. Alonzo Garcelon, Lewiston; Hon. L. A. Emery, Professor of Medical Jurisprudence, Honorary Member Medico-Legal Society and Vice-President for Maine, Ellsworth.

Maryland—Dr. Hampson Jones; William Lee Howard, M. D.; Duncan McCalman, M. D., Baltimore.

Massachusetts—Dr. Henry O. Marcy, Boston; Dr. J. H. Mc-

Cullom, South Division Boston City Hospital, of Boston; Dr. Geo. W. Grover, Sheffield.

Michigan—Hon. Henry A. Height, Detroit; John H. Kellogg, M. D., Battle Creek; Clarence A. Lightner, Esq., Detroit.

Minnesota—Dr. H. Longstreet Taylor, St. Paul.

Missouri—Dr. C. Bark, Dr. Leonidas H. Laidley, both of St. Louis; Dr. A. W. McAlester, Columbia; J. Wood Fassett, M. D., St. Joseph.

Montana—John C. Clayberg, Esq., Helena; John A. Donovan, M. D., Butte; Donald Campbell, M. D., Butte; Thos. J. Murray, M. D., Butte.

Nebraska—Dr. Wm. O. Bridges, 302 Bee Building, Omaha; Dr. Emmett Giffen, Richards Block, Lincoln; Elmer J. Burkett, Esq., Lincoln.

New Hampshire—Dr. G. P. Conn, Concord; Hon. J. W. Fellows, Manchester.

New Jersey—Fred L. Hoffman, Esq., Newark; Ex-Senator H. D. Winton, Hackensack.

New Mexico — Dr. Francis Crosson, Albuquerque; Dr. C. G. Duncan, Socano; Surgeon E. N. Carrington, M. H. S., Fort Stanton, delegate from the Marine Hospital Service, U. S. Government.

New York—Clark Bell, Esq., LL. D., New York City; Dr. Ernst J. Lederle, Ph. D., Ex-President of the Board of Health of the City of New York; Dr. John Vanderpoel, 36 West Thirty-ninth St., New York City; Eugene Porter, M. A., M. D., 171 West Seventy-third St., New York; Leigh Hunt, M. D., 15 East Twelfth St., New York; Roger Foster, Esq., New York City; Hon. Wm. W. Goodrich, Ex-President Justice Appellate Division, Second Department, New York Supreme Court, Vice-Presidents for New York.

North Carolina — Dr. A. W. McAlister, Columbia; Dr. J. L. Nicholson, Richland; Dr. William L. Dunn, Asheville.

North Dakota—Dr. Dwight Shumway Moore, Jamestown.

Ohio—Hon. Joseph Outhwaite, President Ohio Society for the Prevention of Tuberculosis, Columbus.

Oregon—Dr. August C. Kinney; Dr. Andrew C. Smith, President State Board of Health, Portland; Dr. Albert R. Sweetsor, of Eugene, State Biologist, and of the Biological Laboratory of the University of Oregon.

Pennsylvania — Dr. J. W. C. O'Neal, Gettysburg; Dr. M. K. Kassabian, Philadelphia; Major James Evelyn Pilcher, U. S. A., L. H. D., Carlisle, Pa.

Rhode Island—Charles V. Chapin, M. D., Providence; Dr. Geo. F. Keesey, Howard.

South Carolina—George R. Dean, M. D., Spartanburg.

Tennessee—Dr. J. D. Plunkett, Nashville; R. F. Spragins, Esq., Jackson.

Texas—M. M. Smith, M. D., Austin; Yancy Lewis, M. D., Dallas; Dr. J. P. Sessions, Rockdale; Dr. Samuel R. Burroughs, Buffalo; Dr. John T. Moore, Galveston; Dr. T. J. Bennett, Austin.

Utah—Dr. Martha Hughes Cannon, Salt Lake City; Dr. H. A. Anderson, Salt Lake City; Dr. Geo. W. Baker, Ogden; Col. Willard Young, Salt Lake City.

Vermont—Dr. C. S. Caverly, Rutland; Hon. Fletcher Proctor, Proctor; Dr. M. J. Wiltsse, Burlington.

Virginia—George Ben Johnson, M. D., Richmond; Dr. W. F. Drewry, Petersburg; Dr. Paulus A. Irving, Richmond.

Washington, D. C.—Surgeon Preston H. Bailhache, U. S. M. H., Delegate from the U. S. Government; Surgeon W. C. Brasted, U. S. Navy, Delegate from the U. S. Government; Captain Henry D. Snyder, M. D., U. S. Army, Delegate from the U. S. Government to the Congress, Washington, D. C.

Washington—Sofus B. Nelson, Veterinarian, Washington Agricultural Experiment Station, Pullman; Hon. I. M. Byrne, Mayor of Spokane; Dr. Parke Winke, Seattle.

West Virginia—Wm. W. Golden, Elkins, Secretary Medical Society of the State of West Virginia; T. L. Barber, M. D., Charleston; I. N. Houston, M. D., Mountsville.

Wisconsin—Hon. Alvin C. Brager, Milwaukee; Dr. G. A. Riche,

788 College Avenue, Appleton; Dr. Moses J. White, Milwaukee; Dr. Charles Oviatt, Oshkosh; Dr. Hugo Philler, Waukesha.

Wyoming — John W. Lacey, Esq., Cheyenne; Dr. R. Harvey Reed, Rock Spring; Dr. G. G. Verbryck, Cambria.—*Medico-Legal Journal.*

